# Urgent need to reevaluate the latest World Health Organization guidelines for toxic inorganic substances in drinking water

**DOI:** 10.1186/s12940-015-0050-7

**Published:** 2015-08-13

**Authors:** Seth H. Frisbie, Erika J. Mitchell, Bibudhendra Sarkar

**Affiliations:** 1 grid.261219.f000000012160010XDepartment of Chemistry and Biochemistry, Norwich University, Northfield, VT USA; 2 grid.17063.33Department of Molecular Structure and Function, The Research Institute of The Hospital for Sick Children, University of Toronto, Toronto, ON Canada; 3 grid.17063.33Department of Biochemistry, University of Toronto, Toronto, ON Canada

**Keywords:** Drinking-water guidelines, World Health Organization, Inorganics, Trace metals, Public health

## Abstract

The World Health Organization (WHO) has established guidelines for drinking-water quality that cover biological and chemical hazards from both natural and anthropogenic sources. In the most recent edition of *Guidelines for Drinking-water Quality* (2011), the WHO withdrew, suspended, did not establish, or raised guidelines for the inorganic toxic substances manganese, molybdenum, nitrite, aluminum, boron, nickel, uranium, mercury, and selenium. In this paper, we review these changes to the WHO drinking-water guidelines, examining in detail the material presented in the WHO background documents for each of these toxic substances. In some cases, these WHO background documents use literature reviews that do not take into account scientific research published within the last 10 or more years. In addition, there are instances in which standard WHO practices for deriving guidelines are not used; for example, rounding and other mathematical errors are made. According to published meeting reports from the WHO Chemical Aspects Working Group, the WHO has a timetable for revising some of its guidelines for drinking-water quality, but for many of these toxic substances the planned changes are minimal or will be delayed for as long as 5 years. Given the limited nature of the planned WHO revisions to the inorganic toxic substances and the extended timetable for these revisions, we suggest that governments, researchers, and other stakeholders might establish independent recommendations for inorganic toxic substances and possibly other chemicals to proactively protect public health, or at the very least, revert to previous editions of the *Guidelines for Drinking-water Quality*, which were more protective of public health.

## Introduction

The World Health Organization (WHO) has established guidelines for drinking-water quality that cover biological and chemical hazards from both natural and anthropogenic sources. As an organization with worldwide scope and resources, not tied to the political or financial interests of any one country or region, the WHO is uniquely able to provide unbiased guidelines for the international community. WHO guidelines are not regulations, but may be used by governments or other stakeholders for setting local standards. Some countries, especially those with limited resources, use WHO guidelines as *de facto* standards. Therefore, these guidelines often directly affect the most disadvantaged populations. WHO guidelines are also used by researchers when comparing drinking water across regions or countries since the guidelines provide a uniform international measure of water quality, whereas local standards may vary by jurisdiction.

In the most recent edition of *Guidelines for Drinking-water Quality* (2011), the WHO withdrew, suspended, did not establish, or raised guidelines for the inorganic toxic substances manganese, molybdenum, nitrite, aluminum, boron, nickel, uranium, mercury, and selenium [1]. In each case, careful study of the WHO background documents suggests these changes may not adequately protect public health. In contrast, the WHO did not establish any new guidelines for the 62 of 76 (82 %) elements in the earth’s crust that do not currently have a WHO drinking water guideline [1, 2]. The WHO also did not decrease the guidelines for any inorganic compounds [1].

In this paper, we review the 2011 changes to the WHO drinking-water guidelines for the inorganic toxic substances manganese, molybdenum, nitrite, aluminum, boron, nickel, uranium, mercury, and selenium. These inorganic compounds are commonly found in drinking water, through the result of either natural processes or anthropogenic contamination. For example, we evaluate the WHO’s decision not to establish a health-based drinking-water guideline for aluminum, the third most abundant element in the earth’s crust [1, 2].

We do not evaluate changes to the WHO drinking-water guidelines for pesticides and other anthropogenic chemicals. These compounds are not naturally occurring and are only found in polluted drinking water. Thus, we do not evaluate the WHO’s decision to discontinue a health-based drinking-water guideline for cyanide because it “Occurs in drinking-water at concentrations well below those of health concern, except in emergency situations following a spill to a water source” [3], and we do not evaluate the WHO’s decision to discontinue a health-based drinking-water guideline for chlorobenzene because of its taste and odor [1, 3].

The WHO Chemical Aspects Working Group meets several times a year to plan revisions to the WHO drinking-water quality guidelines and to set a timetable for these revisions [3–5]. According to minutes of these meetings [3–5], revisions to some of the drinking-water guidelines for inorganic substances are planned. However, many of the planned revisions are limited in nature and the published timetable for these revisions is extended as late as 2020. Thus, stakeholders may find it prudent to establish independent recommendations for inorganic toxic substances to proactively protect public health, or at the very least, revert to previous editions of the *Guidelines for Drinking-water Quality*, which were more protective of public health.

### Manganese

In 2011 the 400 μg/L drinking-water guideline for manganese was withdrawn with the assertion that since “this health-based value is well above concentrations of manganese normally found in drinking-water, it is not considered necessary to derive a formal guideline value” [1]. However, over 50 countries have drinking-water or potential drinking-water supplies with manganese concentrations above 400 μg/L [6]. In Bangladesh alone, over 60,000,000 people are likely drinking water with manganese above 400 μg/L [6]. Two years after the manganese guideline was withdrawn, WHO staff and consultants wrote a report from the 2013 World Health Organization Meetings on the *Guidelines for Drinking-water Quality* that observed, “There is a lot of concern from developing countries about WHO withdrawing its GV [guideline value] for manganese because it is present at high levels in solution in some community groundwater supplies” [3].

This report noted, “There is an internal inconsistency in the GDWQ [*Guidelines for Drinking-water Quality*]: on p. 387, it states that a GV [guideline value] for manganese is not necessary because it is “Not of health concern at levels found in drinking-water”, whereas on p. 471, it states that manganese is “Not of health concern at levels causing acceptability problems in drinking-water”. The latter statement is more correct, especially considering the high levels of manganese that have been found in drinking-water sources” [3]. In other words, since manganese might affect the taste of drinking-water and stain laundry and plumbing fixtures at less than 400 μg/L, the WHO assumes that a person will not drink such water, so a 400 μg/L guideline is not needed. According to WHO plans [3], for some chemicals, acceptability problems may justify the withdrawal of formal guideline values that are currently based on adverse health effects in the next edition of *Guidelines for Drinking-water Quality*.

However, taste, staining, and other acceptability problems such as odor are subjective, imprecise, and hard to quantify. For example, the WHO states that manganese at 100 μg/L “imparts an undesirable taste to beverages and stains plumbing fixtures and laundry” [7], at 50 μg/L “discoloration may occur” [7], and at 20 μg/L forms “coatings on water pipes that may later slough off as a black precipitate” [7]. These differing values make it unclear which concentration is the most appropriate to choose for an acceptability threshold in place of a health-based guideline value.

**Fig. 1:**
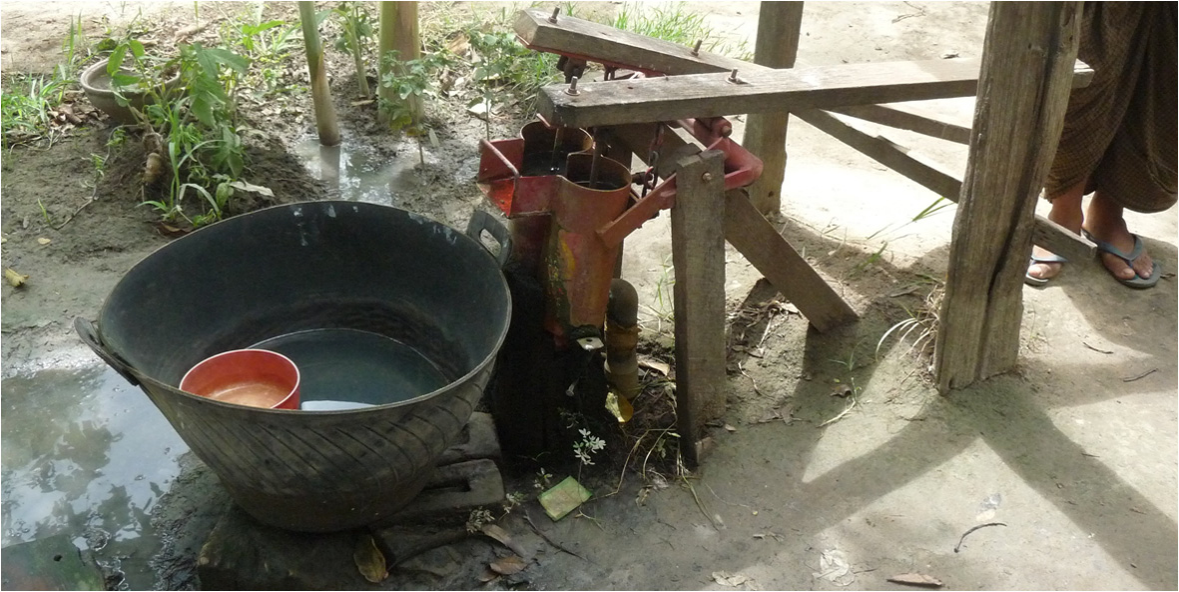
A drinking-water well from Tha Pyay Thar, Myanmar where the minimum, average, and maximum concentration of manganese is 390 μg/L, 1000 μg/L, and 1700 μg/L, respectively. (Photograph by Manja Uhlig, 2013; reproduced by permission of Manja Uhlig)

Furthermore, the assumption that a person will not drink water if the concentration of a chemical is greater than an acceptability threshold may not protect public health. For example, over 60,000,000 people in Bangladesh were found to have been drinking water with an average 940 μg/L of manganese for 6 years in 1998 [8]. In another study, people in western Bangladesh were found to have been drinking water with 400 μg/L to 2400 μg/L of manganese for an average of 9 years in 2002 [9]. People from Bongaon, West Bengal, India have been drinking water with an average, and maximum manganese concentration of 240 μg/L, and 640 μg/L, respectively [10]. People from Tha Pyay Thar, Myanmar have been drinking water with a minimum, average, and maximum manganese concentration of 390 μg/L, 1000 μg/L, and 1700 μg/L, respectively [11]. A photograph of a typical drinking-water plumbing fixture from Tha Pyay Thar is shown in Fig. 1; it is not apparent that the potential for manganese to stain such fixtures may be a concern for the Tha Pyay Thar residents. The fact that tens of millions of people readily drink water with manganese concentrations far above a presumed acceptability threshold highlights the inadvisability of relying on such acceptability thresholds instead of establishing formal health-based guideline values. In conclusion, relying on acceptability thresholds instead of health-based drinking-water guidelines for manganese or other toxic chemicals is not protective of public health and may be especially harmful to the disadvantaged.

The WHO 2011 decision to withdraw the drinking-water guideline for manganese was based on a literature review that did not include any references on human toxicity published after 2001; some studies suggest the former 400 μg/L guideline may have been too high to protect public health [12]. Numerous papers published since 2001 have investigated adverse health effects due to manganese.

Exposure to high levels of manganese has long been recognized to have neurotoxic effects in adults, with excessive manganese exposure linked to Manganism, Parkinsons Disease, and movement disorders [13–17]. Elevated levels of manganese in blood, hair or nails have also been associated with compulsive behaviors, emotional lability, hallucinations, attention disorders, and cognitive decline in adults [18–23] as well as Amyotrophic Lateral Sclerosis (ALS) [24].

A growing body of evidence points to adverse effects of excessive manganese on children’s behavior and intellectual development [25, 26]. A 2009 review by Menezes-Filho et al. [27] of adverse effects of manganese exposure on neurodevelopment in children discusses 5 articles published before 2000 and 7 articles published between 2000 and 2009, while two 2013 reviews by Rodríguez-Barranco et al. [28] and Zoni and Lucchini [29] discuss 10 additional articles on adverse effects of manganese on children’s neurodevelopment published between 2009 and 2012. All of these reviews as well as 2 subsequent reviews by Polańska et al. [30] and Grandjean and Landrigan [31] note that manganese has been linked to behavioral problems, learning disabilities and intellectual deficits in children. Recent papers published in 2013 and 2014 reporting adverse effects of manganese on children’s neurodevelopment include Lin et al. [32], Oulhote et al. [33], Roberts et al. [34], and Torres-Agustín et al. [35]. In contrast, a small study of children with relatively low exposures to manganese found no clear evidence between hair manganese and children’s developmental scores [36]. Also, a small study of the metals content of shed primary teeth found no significant differences in tooth manganese levels between children showing high levels of disruptive behavior compared to typically developing children, but children with autism spectrum disorders showed marginally lower tooth manganese levels than typically developing children [37].

As an essential element, either an excess or a deficiency of manganese has been shown to adversely affect birth outcomes. Elevated levels of cord blood manganese have been found with preeclampsia cases [38]. High levels of manganese exposure have been associated with low birth weight [39, 40]. Other studies have found both low and high levels of maternal or cord blood manganese associated with low birth weight [41–43] or high ponderal index [44]. Both low and high levels of maternal manganese have been found to inhibit calcium-pump activity at birth [45]. In some studies well water with high levels of manganese has been associated with increased infant mortality [46, 47]; other studies found no effects or decreased infant mortality [48–50].

Manganese also affects hormones and the endocrine system. Elevated manganese levels have been indicated as a potential factor in male infertility [51–53]. High manganese levels have also been associated with elevated prolactin at birth [54] and in children ages 7–11 [55]. Both teenagers and adults with goiter have been found to have high levels of manganese [56, 57].

Some individuals may be especially sensitive to manganese exposure. Blood manganese levels tend to be higher with lower blood iron levels [58], and iron deficiency, which is especially common in the developing world, can exacerbate manganese toxicity [59–61]. Individual cases of childhood Manganism [62] or epileptic seizures [63] have been linked to manganese exposure, while excessive manganese accumulation has been implicated in hepatocerebral syndrome [64].

At the 2013 World Health Organization Meetings on the *Guidelines for Drinking-water Quality*, it was decided “If Member States express concern about manganese, WHO will explain that the scientific basis of the manganese HBV [Health-based Value] is under review and that the review is expected to be complete in time for the second addendum.” The second addendum to the 4th edition of the *Guidelines for Drinking-water Quality* is not expected until late 2017 [3]. Given the common occurrence of manganese in drinking-water, the WHO’s decision to rely on an acceptability threshold rather than a formal health-based guideline value, the numerous adverse health effects associated with manganese exposure, and the expected lengthy timetable for possible revisions to the WHO guideline, stakeholders might choose to develop their own independent health-based drinking-water standards for manganese or, at the very least, continue using the former WHO guideline of 400 μg/L published in the previous (3rd) edition of the *Guidelines for Drinking-water Quality* [65].

### Molybdenum

Similarly, in 2011 the 70 μg/L WHO health-based drinking-water guideline for molybdenum was withdrawn with the assertion that molybdenum “occurs in drinking-water at concentrations well below those of health concern”; therefore, “it is not considered necessary to set a formal guideline value” [1]. However, the WHO previously stated that in a “survey of 380 finished water samples from across the USA, 29.9 % contained measurable concentrations of molybdenum, with a mean of 85.9 µg/litre and a range of 3-1024 µg/litre” [66]. The WHO also explained that, “Molybdenum was present in 32.7 % of surface water samples from 15 major river basins in the United States of America (USA) at concentrations ranging from 2 to 1500 μg/l (mean 60 μg/l) (Kopp & Kroner, 1967; National Academy of Sciences, 1977). Levels in groundwater ranged from undetectable to 270 μg/l in another survey in the USA (Kehoe, Chalak & Largent, 1944)” [67]. In addition to the United States [66, 68, 69], molybdenum has been reported in drinking water and potential drinking water supplies at concentrations above the former 70 μg/L WHO health-based guideline in Argentina [70–72], Burkina Faso [73], Cambodia [74], Ethiopia [75, 76], and Iran [77]. Thus, it is not uncommon for molybdenum in drinking water to exceed the former 70 μg/L health-based guideline, so the guideline should be reinstated.

The WHO does not plan to reinstate a guideline for molybdenum before the 5th edition of *Guidelines for Drinking-water Quality*, scheduled for publication in late 2020 [3]. Therefore, stakeholders might choose to develop their own independent health-based drinking-water standards for molybdenum or, at the very least, continue using the former WHO guideline of 70 μg/L published in the previous (3rd) edition of the *Guidelines for Drinking-water Quality* [65].

### Nitrite

In 2011 the 3000 μg/L drinking-water guideline for acute or short-term exposure to nitrite was maintained; however, the 200 μg/L drinking-water guideline for chronic or long-term exposure to nitrite was “suspended and is under review owing to significant uncertainty surrounding the endogenous formation of nitrite and concentrations in human saliva” [1]. In this same document, the WHO recommended that the “average exposures [of nitrite] over time should not exceed about 0.2 mg/l [200 μg/L]” [1]. Given this recommendation, the decision to suspend the 200 μg/L guideline seems inconsistent.

The suspension of the guideline was especially unexpected given the many common sources of chronic exposure to nitrite. According to the WHO, “Nitrite can also be formed chemically in distribution pipes by *Nitrosomonas* bacteria during stagnation of nitrate-containing and oxygen-poor drinking-water in galvanized steel pipes or if chloramination is used to provide a residual disinfectant” [1]. In addition, the use of septic tanks and cesspools to dispose of human excrement, and the use of nitrogen fertilizers and animal manures by agriculture are common sources of nitrite in drinking water [78].

The WHO plans to issue a revised background document and summary statement for nitrate/nitrite in late 2015 [3]. It is unclear whether a guideline for chronic exposure to nitrite will be reinstated at that time. Until a formal guideline for chronic exposure to nitrite is reinstated, stakeholders might decide to develop their own independent health-based drinking-water standards for the chronic exposure to nitrite or, at the very least, follow the WHO’s recommendation that the “average exposures [of nitrite] over time should not exceed about 0.2 mg/l [200 μg/L]” [1].

### Aluminum

In 2011 the WHO stated that a 900 μg/L health-based drinking-water guideline for aluminum “could be derived”; nevertheless, this guideline was not applied because it exceeds the expected concentration of aluminum remaining in drinking water after treatment with aluminum-based coagulants [1]. However, treatment with aluminum-based coagulants is not the only source of aluminum in drinking water.

For example, drinking water with ambient aluminum concentrations above 900 μg/L has been reported in the United States, according to the WHO [66]. Similarly, drinking water and potential drinking water supplies with ambient aluminum concentrations above 900 μg/L have been reported in Argentina [71, 72], Bangladesh [79], Brazil [80, 81], Ethiopia [76], Ghana [82], India [83], Iran [77], Kosovo [84], Lesotho [85], Sri Lanka [86], Sweden [87], Tanzania [88], Turkey [89], and Uganda [90]. The WHO noted that, “The concentration of aluminium in natural waters can vary significantly depending on various physicochemical and mineralogical factors. Dissolved aluminium concentrations in waters with near-neutral pH values usually range from 0.001 to 0.05 mg/l [1 μg/L to 50 μg/L] but rise to 0.5–1 mg/l [500 μg/L to 1000 μg/L] in more acidic waters or water rich in organic matter. At the extreme acidity of waters affected by acid mine drainage, dissolved aluminium concentrations of up to 90 mg/l [90,000 μg/L] have been measured” [91].

Thus, untreated drinking water can contain aluminum, either from natural or anthropogenic sources, at concentrations high enough to cause adverse health effects. Drinking-water guidelines are used to determine safety, and as a result, must apply to both natural and anthropogenic sources of contamination. Therefore, a health-based guideline for aluminum is vital for determining the safety of untreated drinking water.

### Boron

In 2011 the WHO increased the drinking-water guideline for boron from 500 μg/L to 2400 μg/L [1]. Notably, this 2400 μg/L guideline is approximately 20 % high due to a rounding error in an intermediate step of the calculation, contradicting WHO policy [1]. The 2013 World Health Organization Meetings on the *Guidelines for Drinking-water Quality* confirmed that, “The GV [guideline value] or HBV [health-based value] for the GDWQ [*Guidelines for Drinking-water Quality*] will be rounded at the final step only. This will need to be clearly explained in the guideline derivation section” of the WHO *Policies and Procedures Manual* [3].

Specifically, the tolerable daily intake (TDI) of boron was rounded up during an intermediate step from 0.17 mg/kg to 0.2 mg/kg of body weight [92]. The WHO explained that, “Applying an uncertainty factor of 60 to the BMDL_05_ [a lower 95 % confidence limit on the benchmark dose] of 10.3 mg/kg body weight gives a TDI of 0.17 mg/kg body weight, rounded to 0.2 mg/kg body weight” [92]. The calculation of TDI is shown in Equation 1.

1 $$ \mathrm{T}\mathrm{D}\mathrm{I}\kern0.5em =\kern0.5em \frac{\left(\frac{10.3\kern0.2em \mathrm{mg}\kern0.2em \mathrm{of}\kern0.2em \mathrm{boron}}{\mathrm{kg}\kern0.2em \mathrm{of}\kern0.2em \mathrm{body}\kern0.2em \mathrm{weight}\times \mathrm{day}}\right)}{60\kern0.5em \mathrm{uncertainty}\kern0.5em \mathrm{factor}}=\frac{0.17\kern0.5em \mathrm{mg}\kern0.5em \mathrm{of}\kern0.5em \mathrm{boron}}{\mathrm{kg}\kern0.5em \mathrm{of}\kern0.5em \mathrm{body}\kern0.5em \mathrm{weight}\kern0.5em \times \kern0.5em \mathrm{day}} $$

The current 2400 μg/L guideline based on the incorrectly rounded TDI of 0.2 mg/kg body weight is shown in Equations 2 and 3. This calculation assumes that a 60 kg person drinks 2 L of water per day, and that 40 % of the ingested boron is from this drinking water [92].

2 $$ \mathrm{Guideline}\kern1em =\kern0.5em \frac{0.2\kern0.5em \mathrm{mg}\kern0.5em \mathrm{of}\kern0.5em \mathrm{boron}}{\mathrm{kg}\kern0.5em \mathrm{of}\kern0.5em \mathrm{body}\kern0.5em \mathrm{weight}\kern0.5em \times \kern0.5em \mathrm{day}}\kern0.5em \times \kern0.5em \frac{60\kern0.5em \mathrm{kg}\kern0.5em \mathrm{of}\kern0.5em \mathrm{body}\kern0.5em \mathrm{weight}}{\left(\frac{2\kern0.2em \mathrm{L}\kern0.2em \mathrm{of}\kern0.2em \mathrm{drinking}\kern0.2em \mathrm{water}}{\mathrm{day}}\right)}\kern0.5em \times \kern0.5em 40\% $$

3 $$ \mathrm{Guideline}\kern1em =\kern1em \frac{2.4\kern0.5em \mathrm{mg}\kern0.5em \mathrm{of}\kern0.5em \mathrm{boron}}{\mathrm{L}\kern0.5em \mathrm{of}\kern0.5em \mathrm{drinking}\kern0.5em \mathrm{water}}\kern0.5em =\kern0.5em \frac{2,400\kern0.5em \upmu \mathrm{g}\kern0.5em \mathrm{of}\kern0.5em \mathrm{boron}}{\mathrm{L}\kern0.5em \mathrm{of}\kern0.5em \mathrm{drinking}\kern0.5em \mathrm{water}} $$

Without this rounding error, the guideline for boron would have been 2000 μg/L. Using the unrounded TDI of 0.17 mg/kg body weight would yield a corrected guideline of 2000 μg/L, as shown in Equations 4 and 5. Note that guidelines are “usually rounded to one significant figure” [1] during the final step of calculation, according to the WHO.

4 $$ \mathrm{Guideline}\kern0.5em =\kern0.5em \frac{0.17\kern0.5em \mathrm{mg}\kern0.5em \mathrm{of}\kern0.5em \mathrm{boron}}{\mathrm{kg}\kern0.5em \mathrm{of}\kern0.5em \mathrm{body}\kern0.5em \mathrm{weight}\kern0.5em \times \kern0.5em \mathrm{day}}\kern0.5em \times \kern0.5em \frac{60\kern0.5em \mathrm{kg}\kern0.5em \mathrm{of}\kern0.5em \mathrm{body}\kern0.5em \mathrm{weight}}{\left(\frac{2\kern0.2em \mathrm{L}\kern0.2em \mathrm{of}\kern0.2em \mathrm{drinking}\kern0.2em \mathrm{water}}{\mathrm{day}}\right)}\kern0.5em \times \kern0.5em 40\% $$

5 $$ \mathrm{Guideline}\kern0.5em =\kern0.5em \frac{2\kern0.5em \mathrm{mg}\kern0.5em \mathrm{of}\kern0.5em \mathrm{boron}}{\mathrm{L}\kern0.5em \mathrm{of}\kern0.5em \mathrm{drinking}\kern0.5em \mathrm{water}}\kern0.5em =\kern0.5em \frac{2,000\kern0.5em \upmu \mathrm{g}\kern0.5em \mathrm{of}\kern0.5em \mathrm{boron}}{\mathrm{L}\kern0.5em \mathrm{of}\kern0.5em \mathrm{drinking}\kern0.5em \mathrm{water}} $$

The 2013 World Health Organization Meetings on the *Guidelines for Drinking-water Quality* did not specifically identify this rounding error for boron [3]. It is essential that the WHO correct this rounding error; in the meantime, stakeholders should establish health-based drinking-water standards for boron that do not include rounding errors.

### Nickel

In 2006 the WHO increased the health-based drinking-water guideline for nickel from 20 μg/L to 70 μg/L [93]. This increase was maintained in 2011 [1]. The former 20 μg/L guideline was based on several peer-reviewed studies of rats, mice, and dogs that found adverse kidney, liver, and reproductive effects from chronic exposure to nickel [94]. When developing the current 70 μg/L guideline, instead of relying on these several peer-reviewed studies, the WHO selected a single, more recent, unpublished, industry-funded 2-generation study of chronic exposures in rats that was performed by a commercial laboratory [95]; an interlibrary loan request for this unpublished study was declined by the industry group. In contrast to the several peer-reviewed studies that gave a 20 μg/L guideline, this single, unpublished, industry-funded study suggested a 130 μg/L guideline. Somewhat unexpectedly, an acute exposure study producing eczema in humans yielded a lower guideline than this unpublished chronic exposure study, so the current 70 μg/L guideline protects against eczema as an acute exposure effect and discounts possible health effects due to chronic exposures [95].

It is unclear why a lower 20 μg/L guideline that protects against a more likely chronic exposure was replaced with a higher 70 μg/L guideline that protects against a less likely acute exposure. This example for nickel may be the first time in the history of the WHO that a relatively low guideline for a chronic exposure was replaced with a relatively high guideline for an acute exposure. Some of the attendees at the 2013 World Health Organization Meetings on the *Guidelines for Drinking-water Quality* expressed concern about replacing a guideline that is based on chronic exposure with a guideline that is based on acute exposure: “Another concern is that the WHO GV [guideline value] is based on a LOAEL [lowest observed adverse effect level] for an acute effect” [3]. At the meeting it was concluded that, “Guidance on deriving GVs based on an acute effect is needed” [3] and “A robust evaluation needs to be undertaken” [3]. However, no deadline for this evaluation was scheduled [3].

Of further concern is that, when revising the nickel guideline, the WHO increased the allocation for exposure from drinking water from 10 to 20 %, effectively doubling the guideline [94, 95]. The former 10 % allocation was based on a peer-reviewed article and a government report [94]; the WHO did not provide references supporting this allocation increase for nickel [95]. It is possible that this increase from 10 to 20 % might derive from the following change to the WHO *Policies and Procedures Manual*, “In most circumstances, allocation factors will range from 20 % to 80 % (default allocation factors are 20 % for chemicals for which drinking-water is not the main exposure route and 80 % for chemicals for which drinking-water is the main exposure route)” [3]. If so, it would be more protective of public health to replace the default 20 % allocation for nickel exposure from drinking water with the peer-reviewed 10 % allocation. The report from the 2013 World Health Organization Meetings on the *Guidelines for Drinking-water Quality* would seemingly support this return to a more protective 10 % value, “Based on exposure patterns, it may be necessary to revisit the allocation factor used in the derivation of the WHO GV [guideline value] for nickel”; however, the “time frame [for this reevaluation is] unknown” [3].

### Uranium

In 2011 the WHO increased the provisional drinking-water guideline for uranium from 15 μg/L to 30 μg/L [1, 96, 97]; the WHO notes “The guideline value is designated as provisional because of scientific uncertainties surrounding uranium toxicity…as well as difficulties concerning its technical achievability in smaller supplies” [1]. The 30 μg/L health-based guideline was calculated using a “no-effect group” with “no evidence of renal damage” based on a 2006 study of human adults who drank water with a median uranium concentration of 25 μg/L for an average of 16 years [96–98]. However, this nominal “no-effect group” showed statistically significant increases in diastolic blood pressure, systolic blood pressure, and glucose excretion in urine [98]. The study authors note that for the increased blood pressure, “the effect was small and no clear hypertension was observed” [98]. However, they also point out “The biomarkers chosen for this study, if measured at a single point in time, may not adequately identify renal injury from long-term exposure because, for example, the kidney might adapt to long-term stable exposures such that there is a decrease in the ability of proximal tubular cells to excrete these markers into urine” [98]. It must be kept in mind that this “no-effect group” was a subpopulation from a larger 2002 study by Kurttio et al. that showed statistically significant increases in calcium fractional excretion, phosphate fractional excretion, diastolic blood pressure, systolic blood pressure, and diuresis [96, 98, 99]. Another study published in 2005 by Kurttio et al. used a different subpopulation from the 2002 Kurttio et al. study consisting of 146 men and 142 women who drank well water with a median uranium concentration of 27 μg/L for an average of 13 years [100]. This 2005 Kurttio et al. study found increased levels of bone turnover markers with increased uranium exposure in men [100]. These results showing potential adverse effects in the 3 related Kurttio et al. studies [98–100] put into question whether the Kurttio et al. 2006 group can appropriately be termed a “no-effect” group [96].

Although the term “benchmarking” does not appear in the WHO’s background document explaining the rationale for raising the guideline for uranium [97], the method of calculating the current 30 μg/L guideline bears some resemblance to a benchmarking approach. The WHO note that “When appropriate data for mathematical modelling of dose–response relationships are available, BMDLs [Lower Confidence Limit on the Benchmark Dose] are used as alternatives to NOAELs in the calculation of health-based guideline values” [1]. The WHO define a BMDL as “the lower confidence limit of the dose that produces a small increase (e.g. 5 % or 10 %) in the level of adverse effects” [1]. In the derivation of the 2011 uranium guideline, the WHO state “Considering the Kurttio et al. (2006a) study group as a no-effect group, the value of the 95th percentile of the uranium exposure distribution is considered to be a NOAEL. From the analysis of the uranium exposure of approximately 200 people, the value of the 95th percentile is estimated to be 1094 μg/day, and the 95 % confidence interval is calculated to be 637–1646 μg/day using a bootstrap method. The lower confidence limit of 637 μg/day is appropriate for the point of departure” [97]. This reference to a lower confidence limit of a dose is somewhat suggestive of benchmarking techniques. However, since the WHO assume there were no adverse effects in the Kurttio et al. 2006 population, there could be no evidence for a dose that produces a small increase in effects, and the WHO also do not discuss the existence of a dose–response curve for the Kurttio et al. 2006 population [97]. Thus, despite the reference to the lower confidence limit of a dose, the WHO appear to be taking a NOAEL approach in calculating this guideline rather than benchmarking.

By definition, a NOAEL is “the highest dose or concentration of a chemical in a single study, found by experiment or observation, that causes no detectable adverse health effect” [1]. To reduce the uncertainty of potential interspecies variation, human exposure studies are preferred when available [1]. For ethical reasons, toxicological studies involving human populations are typically epidemiological studies, in which a sample group has been exposed accidentally to a toxic substance, often with a wide range of exposure concentrations. Some such studies divide the sample population into discrete exposures groups and the groups are compared to see if any statistically different patterns of effects may be discerned (e.g. [101, 102]). Alternatively, conclusions are drawn from the exposed population as a whole as compared to either defined reference values for tested parameters or a control population; in these cases, the mean or median exposure concentration of the undifferentiated exposed population is generally used for the comparison [66, 103].

In the 2006 Kurttio et al. study of human uranium exposure on which the 2011 WHO guideline is based, a total of 193 persons drank water containing from 0.03 to 1500 μg/L uranium, with a median exposure of 25 μg/L (36 μg/day) across the entire group, and an interquartile range of 5 to 148 μg/L [98]. Perhaps due to the relatively small sample size, Kurttio et al. 2006 did not separate the total population into distinct exposure groups for the purposes of statistical analyses. However, in an earlier larger study of 325 people, of which the 2006 study population was a subset [99], the authors did divide the population into discrete exposure groups, and they noted “We observed a statistically significant increase in phosphate fractional excretion for drinking water uranium concentration > 300 μg/L relative to < 2 μg/L…. Similarly, the study persons with the highest uranium excretion and intake had elevated calcium and phosphate fractional excretion compared with the lowest exposure groups.... Uranium exposure was associated with increased systolic and diastolic blood pressures and diuresis (urine volume/time) when continuous exposure variables were used…, but the association was statistically significant only between diuresis and the highest categoric exposure group (uranium in urine) compared with the lowest exposure group” [99]. Such results suggest that, given a larger population of highly exposed subjects, it is possible that statistically significant differences might have emerged between high and low exposure subgroups within the 2006 Kurttio et al. study. Thus, it would be inadvisable to assume that lack of effects might be found equally across the entire range of exposures tested by the Kurttio et al. 2006 study.

However, in analyzing the data from the Kurttio et al. 2006 study for use in setting the current drinking-water guideline for uranium, the WHO effectively assumed that no effects were found at the highest exposure levels included in the study. Starting with the high extreme exposure of 1500 μg/L, the WHO first estimated the 95th percentile of the high extreme exposure (1094 μg/day), then used an unspecified bootstrap method to construct a 95 % confidence interval around this 95th percentile (637–1646 μg/day) [96, 97]. They then selected the lower 95 % confidence limit (637 μg/day) of the 95th percentile of exposure as the NOAEL for the study [96, 97]. It must be noted that by constructing a confidence interval around the 95th percentile of exposure, only a small portion of the data are considered, 5 % of the total 193 subjects in the study, or approximately 10 people [96]. Basing a NOAEL on an exposure extreme when the conclusion of no observed adverse effects was drawn from the entire group of exposures, as was done in this case, produces a figure that is likely biased high and not representative of the exposures actually experienced by the study group as a whole [96]. If a guideline is to be derived from the Kurttio et al. 2006 study, given the relatively small size of the study and the fact that discrete exposure groups were not specifically identified or compared statistically by the original study authors, it would be more protective to develop a NOAEL based on a mean, median, or some other measure of central tendency of exposure, a level which a majority of the group actually experienced. Indeed, other WHO guidelines based on epidemiological studies of human exposures typically are based on the mean or median exposure experienced by the group as a whole rather than on an exposure extreme [66, 103]. Notably, the 637 μg/day NOAEL derived by the WHO from the Kurttio et al. 2006 study is approximately 18 times greater than the 36 μg/day median exposure of this nominal no-effect group [98], and yields a 30 μg/L drinking-water guideline that is most likely too high to protect public health [96, 97].

The WHO has commissioned WCA Environment Ltd. to “perform a preliminary assessment of the gaps in the evidence base to support the potential derivation or update of guidelines for 29 substances”, including uranium [3]. At the 2013 Chemical Aspects Working Group Meeting some participants suggested that further literature review for uranium would not be necessary since the guideline for uranium was recently revised in 2011 “and it is unlikely that there will be new data to consider”, while others suggested that toxicological data for this compound “could be looked at” [3]. Thus, it is unclear at this point whether toxicological data published after 2009, the date of the most recent paper cited by the WHO in setting the 2011 guideline, will be considered in the next edition of the *Guidelines for Drinking-water Quality*.

It should be kept in mind that the WHO note “In rural areas with high natural uranium levels, uranium concentrations lower than the guideline value may be difficult to achieve with the treatment technology available (WRc 1997)…As most exposure is from small supplies for which resources are likely to be limited and alternative supplies that are microbiologically safe may not be readily available, care should be taken in responding to an exceedance of the guideline value, which is probably conservative” [97]. Nevertheless, since effective removal of uranium is possible in many areas with high natural uranium concentrations, it would be prudent to base a guideline for uranium solely on health-based concerns rather than on treatability with the most basic technologies. Treatment technologies are constantly being improved, and may be driven by the guidelines themselves; taking into account limitations of current technologies to remove a contaminant when setting a guideline may curb motivation for developing more effective treatment technologies and improving global public health.

### Mercury

In 2006 the WHO changed the health-based drinking-water guideline for mercury from 1 μg/L of total mercury to 6 μg/L of inorganic mercury [104]. This change was maintained in 2011 [1]. The former 1 μg/L guideline was based on the toxicity of methylmercury, an organic and highly toxic form of mercury found in the environment [105]. To explain why the former guideline for mercury was based on the toxicity of methylmercury, the WHO stated “it is unlikely that there is any direct risk of the intake of organic mercury compounds, and especially of alkymercurials [such as methyl- and ethylmercury compounds], as a result of the ingestion of drinking-water. However, there is a real possibility that methylmercury will be converted into inorganic mercury” [105]. Thus, “To be on the conservative side, the PTWI [Provisional Tolerable Weekly Intake] for methylmercury was used to derive a guideline value for inorganic mercury in drinking-water” [105]. Specifically, the former 1 μg/L guideline was based on a 5 μg of total mercury/kg of body weight PTWI, of which no more than 3.3 μg/kg of body weight should be methylmercury [105]. This PTWI was derived by the Joint FAO/WHO Expert Committee on Food Additives (JECFA) from human health effects found in an adult population in Niigata, Japan [104, 106]. Based on more recent studies of neurological effects in children in the Seychelles and Faroe Islands, in 2004, JECFA lowered the PTWI for methylmercury to 1.6 μg/kg of body weight to protect developing fetuses, the most sensitive population [107, 108]. If the practice of basing the drinking water guideline for total mercury on the PTWI for methylmercury had been continued in 2006, the former 1 μg/L drinking-water guideline would have been lowered significantly to protect developing fetuses.

Instead, in 2006, the WHO revised the drinking-water guideline for mercury so that it only applied to inorganic mercury; there is no longer any drinking-water guideline that applies to organic mercury [93, 104]. The revised guideline is based on the toxicity of inorganic mercury in rats [104]. This guideline is derived using a TDI of 2 μg/kg of body weight, that was recommended by the International Programme on Chemical Safety (IPCS) Working Group in 2003 as shown in Equations 6 and 7 [104, 109]. This calculation assumes that a 60 kg person drinks 2 L of water per day, and that 10 % of the ingested inorganic mercury is from this drinking water.

6 $$ \mathrm{Guideline}\kern0.5em =\kern0.5em \frac{2\kern0.5em \upmu \mathrm{g}\kern0.5em \mathrm{of}\kern0.5em \mathrm{inorganic}\kern0.5em \mathrm{mercury}}{\mathrm{kg}\kern0.5em \mathrm{of}\kern0.5em \mathrm{body}\kern0.5em \mathrm{weight}\kern0.5em \times \kern0.5em \mathrm{day}}\kern0.5em \times \kern0.5em \frac{60\kern0.5em \mathrm{kg}\kern0.5em \mathrm{of}\kern0.5em \mathrm{body}\kern0.5em \mathrm{weight}}{\left(\frac{2\kern0.2em \mathrm{L}\kern0.2em \mathrm{of}\kern0.2em \mathrm{drinking}\kern0.2em \mathrm{water}}{\mathrm{day}}\right)}\kern0.5em \times \kern0.5em 10\% $$

7 $$ \mathrm{Guideline}\kern0.5em =\kern0.5em \frac{6\kern0.5em \upmu \mathrm{g}\kern0.5em \mathrm{of}\kern0.5em \mathrm{inorganic}\kern0.5em \mathrm{mercury}}{\mathrm{L}\kern0.5em \mathrm{of}\kern0.5em \mathrm{drinking}\kern0.5em \mathrm{water}} $$

After conducting a review of the literature through January 1999, the IPCS Working Group based their 2003 TDI on a 1993 National Toxicology Program (NTP) study in rats. A more recent 2011 JECFA reexamination of the same 1993 NTP rat study recommended lowering the PTWI for inorganic mercury to 4 μg/kg of body weight [110], corresponding to a lower TDI for inorganic mercury of 0.6 μg/kg of body weight; this TDI would lead to a lower drinking-water guideline of 2 μg/L as shown in Equations 8, 9, and 10. As in Equations 6 and 7, this guideline calculation assumes that a 60 kg person drinks 2 L of water per day, and that 10 % of the ingested inorganic mercury is from this drinking water.

8 $$ \mathrm{T}\mathrm{D}\mathrm{I}\kern0.5em =\kern0.5em \frac{4\kern0.5em \upmu \mathrm{g}\kern0.5em \mathrm{of}\kern0.5em \mathrm{inorganic}\kern0.5em \mathrm{mercury}}{\mathrm{kg}\kern0.5em \mathrm{of}\kern0.5em \mathrm{body}\kern0.5em \mathrm{weight}\kern0.5em \times \kern0.5em \mathrm{week}}\kern0.5em \times \kern0.5em \frac{1\kern0.5em \mathrm{week}}{7\kern0.5em \mathrm{day}\mathrm{s}}\kern0.5em =\kern0.5em \frac{0.6\kern0.5em \upmu \mathrm{g}\kern0.5em \mathrm{of}\kern0.5em \mathrm{inorganic}\kern0.5em \mathrm{mercury}}{\mathrm{kg}\kern0.5em \mathrm{of}\kern0.5em \mathrm{body}\kern0.5em \mathrm{weight}\kern0.5em \times \kern0.5em \mathrm{day}} $$

9 $$ \mathrm{Guideline}\kern0.5em =\kern0.5em \frac{4\kern0.5em \upmu \mathrm{g}\kern0.5em \mathrm{of}\kern0.5em \mathrm{inorganic}\kern0.5em \mathrm{mercury}}{\mathrm{kg}\kern0.5em \mathrm{of}\kern0.5em \mathrm{body}\kern0.5em \mathrm{weight}\kern0.5em \times \kern0.5em \mathrm{week}}\kern0.5em \times \kern0.5em \frac{1\kern0.5em \mathrm{week}}{7\kern0.5em \mathrm{days}}\kern0.5em \times \kern0.5em \frac{60\kern0.5em \mathrm{kg}\kern0.5em \mathrm{of}\kern0.5em \mathrm{body}\kern0.5em \mathrm{weight}}{\left(\frac{2\kern0.2em \mathrm{L}\kern0.2em \mathrm{of}\kern0.2em \mathrm{drinking}\kern0.2em \mathrm{water}}{\mathrm{day}}\right)}\kern0.5em \times \kern0.5em 10\% $$

10 $$ \mathrm{Guideline}\kern0.5em =\kern0.5em \frac{2\kern0.5em \upmu \mathrm{g}\kern0.5em \mathrm{of}\kern0.5em \mathrm{inorganic}\kern0.5em \mathrm{mercury}}{\mathrm{L}\kern0.5em \mathrm{of}\kern0.5em \mathrm{drinking}\kern0.5em \mathrm{water}} $$

At the 2013 World Health Organization Meetings on the Guidelines for Drinking-water Quality, it was stated that “The Chemical WG [Working Group] noted that consideration should be given to updating the background documents on mercury and cyanide based on the recent JECFA assessments,” but no specific timeline for a possible update to the mercury background document was provided [3].

A further concern for having a drinking-water guideline that applies only to inorganic mercury is the challenge of speciation. Mercury speciation requires advanced laboratory methods and expensive equipment that may not be available in routine drinking water testing laboratories, especially in the developing world [111]. Furthermore, mercury determination, especially speciation, requires special care during sampling and storage in order to achieve accurate results [112]. Often when water is tested for mercury, only total mercury levels are reported [109], and concentrations of specifically inorganic mercury, or the possible occurrence of organic mercury in drinking water are not known (e.g. Nicaragua [113], Pakistan [114], Philippines [115], and Poland [116]). Thus, in areas where mercury speciation is not readily achievable, a drinking-water guideline for only inorganic mercury presents potential difficulties for measuring compliance. If a laboratory is not able to speciate mercury, there is a risk that the guideline of 6 μg/L for inorganic mercury might be applied to total mercury and that possible exposures to organic mercury might be overlooked.

The assumption that a drinking-water guideline for mercury need not take into account the potential for exposures to organic mercury must also be given more consideration. Organic mercury has been found in potable groundwater in the United States [117]. It has also been reported in polluted surface waters in Canada [66], China [118], Ghana [119], India [120], Japan [66], Romania [121], and Sweden [122] at levels that could cause adverse health effects if these waters were ever to be adopted as drinking water sources. For many areas, little is known about the actual occurrence of organic mercury in drinking-water since speciation studies are rarely done. A further issue for relying on speciation for assessing the safety of a water supply is that mercury in the environment is subject to conversion from inorganic to organic forms, and from organic to inorganic forms [123].

Mercury is an extremely potent toxic substance with no biological use in the body [124]. It causes a variety of adverse health effects, including the renal changes found in rats that the current drinking-water guideline for inorganic mercury is based on [93, 125] and the neurological effects in children that a guideline for methylmercury could potentially be based on [107, 108]. In addition, recent research has shown that chronic exposure to low concentrations of mercury has been noted to cause diverse neurological effects in adults [124], and cardiovascular [126], renal [123], visual [127], auditory [128], endocrine [129], immune [123], and reproductive [130] effects in humans, and there is considerable genetic variation in susceptibility to mercury intoxication [130].

Significant groups of people, particularly frequent consumers of fish, have body burdens of total mercury that already exceed safe levels [131–134]. For example, all 720 patients who came for an office visit to a private internal medicine practice in San Francisco, California during a 1 year period were evaluated for mercury risk from their reported fish consumption and symptomology, including fatigue, headache, decreased memory, decreased concentration, and muscle or joint pain. At least 103, or 14 %, of these patients had blood levels greater than or equal to the 5.0 μg/L maximum recommended by the U.S. Environmental Protection Agency (U.S. EPA) and the National Academy of Sciences [132]. Essentially all human exposure to methylmercury is from the consumption of fish, according to the U.S. EPA [135]. As a result, some jurisdictions have chosen to set the source allocation factor for methylmercury in drinking water to 0 [136]. Since methylmercury exposure from the consumption of fish constitutes such a high proportion of total exposure to this toxic substance, and high-end consumers of fish have been shown to develop symptoms of chronic mercury poisoning [132], a 0 % source allocation factor for mercury may be appropriate for deriving a drinking water guideline. Thus, the 10 % source allocation factor for water used by the WHO in deriving the drinking water guideline for mercury may be too high.

Given the difficult nature of mercury speciation in drinking water and the likelihood that water contaminated with mercury may contain some methylmercury [137], stakeholders might prefer to retain the former 2003 WHO guideline of 1 μg/L for total mercury, at least until the WHO is able to update its risk assessment for this toxic substance according to recent JECFA assessments [108, 110] and other research published during the last 10 years.

### Selenium

In 2011 the WHO increased the health-based drinking-water guideline for selenium from 10 μg/L to 40 μg/L [1]. Selenium is an essential trace element, with a recommended daily intake of 0.9 μg/kg of body weight for adults; selenium deficiency may be a co-factor in Keshan disease (endemic cardiomyopathy), Kashin-Beck disease (chronic, degenerative osteoarthropathy), and some autoimmune diseases [103, 138]. In contrast, excessive intake of selenium in humans has been associated with adverse skin, tooth, nail, hair, gastrointestinal, or neurological effects [103]; it has also been implicated with diabetes, Amyotrophic Lateral Sclerosis (ALS), and skin cancer [138–140]. For many aspects of human health, including preventing cardiovascular disease, reducing cancer risk, and increasing reproductive success, response to selenium is often characterized by a U-shaped response curve, with both high and low extremes of serum selenium concentrations associated with adverse outcomes [138]. The former WHO drinking-water guideline of 10 μg/L was calculated from a human epidemiological study in which no adverse health effects were reported for 142 persons with a mean daily selenium intake of 0.24 mg from food [103, 141]. The calculation of the NOAEL for the former guideline is shown in Equations 11 and 12. This calculation assumes that a person weighs 60 kg. The WHO reported a NOAEL of “about 4 μg/kg of body weight per day” to 1 significant figure as follows [103]:

11 $$ \mathrm{NOAEL}\kern0.5em =\kern0.5em \frac{\left(\frac{0.24\kern0.2em \mathrm{mg}\kern0.2em \mathrm{of}\kern0.2em \mathrm{selenium}}{\mathrm{day}}\kern0.5em \times \kern0.5em \frac{1,000\kern0.2em \upmu \mathrm{g}\kern0.2em \mathrm{of}\kern0.2em \mathrm{selenium}}{1\kern0.2em \mathrm{mg}\kern0.2em \mathrm{of}\kern0.2em \mathrm{selenium}}\right)}{60\kern0.5em \mathrm{kg}\kern0.5em \mathrm{of}\kern0.5em \mathrm{body}\kern0.5em \mathrm{weight}} $$

12 $$ \mathrm{NOAEL}\kern0.5em =\kern0.5em \frac{4\kern0.62em \upmu \mathrm{g}\kern0.5em \mathrm{of}\kern0.5em \mathrm{selenium}}{\mathrm{kg}\kern0.5em \mathrm{of}\kern0.5em \mathrm{body}\kern0.5em \mathrm{weight}\kern0.5em \times \kern0.5em \mathrm{day}} $$

This NOAEL of 4 μg/kg body weight per day was used in the calculation of the former 10 μg/L WHO guideline, as shown in Equations 13 and 14. This calculation assumes that a 60 kg person drinks 2 L of water a day, and that 10 % of the ingested selenium is from this drinking water. The final result was rounded to 1 significant figure as follows [103]:

13 $$ \mathrm{Guideline}\kern0.5em =\frac{4\kern0.5em \upmu \mathrm{g}\kern0.5em \mathrm{of}\kern0.5em \mathrm{selenium}}{\mathrm{kg}\kern0.5em \mathrm{of}\kern0.5em \mathrm{body}\kern0.5em \mathrm{weight}\kern0.5em \times \kern0.5em \mathrm{day}}\kern0.5em \times \kern0.5em \frac{60\kern0.5em \mathrm{kg}\kern0.5em \mathrm{of}\kern0.5em \mathrm{body}\kern0.5em \mathrm{weight}}{\left(\frac{2\kern0.2em \mathrm{L}\kern0.2em \mathrm{of}\kern0.2em \mathrm{drinking}\kern0.2em \mathrm{water}}{\mathrm{day}}\right)}\kern0.5em \times \kern0.5em 10\% $$

14 $$ \mathrm{Guideline}\kern0.5em =\kern0.5em \frac{10\kern0.5em \upmu \mathrm{g}\kern0.5em \mathrm{of}\kern0.5em \mathrm{selenium}}{\mathrm{L}\kern0.5em \mathrm{of}\kern0.5em \mathrm{drinking}\kern0.5em \mathrm{water}} $$

In contrast, the current WHO drinking-water guideline of 40 μg/L was calculated from the upper tolerable intake of 400 μg/day of selenium, established in 2000 by the United States National Academy of Sciences [1, 142, 143]. The National Academy of Sciences 400 μg/day Upper Limit (UL) for selenium was based on a series of studies of human selenium exposure in Hubei, China, especially a 1994 study by Yang and Zhou in which 5 patients who previously showed overt symptoms of selenium poisoning (hair loss and nail sloughing) had recovered when their selenium intake decreased to about 800 μg/day, although their nails still reportedly appeared brittle [142, 144]. The calculation of the 400 μg/day UL is shown in Equation 15. This calculation uses an uncertainty factor of 2 as follows [142]:

15 $$ \mathrm{Upper}\kern0.5em \mathrm{Limit}\kern0.5em =\kern0.5em \frac{\left(\frac{800\kern0.2em \upmu \mathrm{g}\kern0.2em \mathrm{of}\kern0.2em \mathrm{selenium}}{\mathrm{day}}\right)}{2\kern0.5em \mathrm{uncertainty}\kern0.5em \mathrm{factor}}=\frac{400\kern0.5em \upmu \mathrm{g}\kern0.5em \mathrm{of}\kern0.5em \mathrm{selenium}}{\mathrm{day}} $$

It should be noted that the 400 μg/day UL calculated by the National Academy of Sciences applies specifically to adults. Although the National Academy of Sciences did not find evidence indicating increased sensitivity for any age group, they did establish separate ULs for different ages based on relative body weights. These ULs are shown in Table 1.

**Table 1:** The Upper Limits (ULs) of selenium for children [142]

Age	Upper limit
0 to 6 months	45 μg/day
7 to 12 months	60 μg/day
1 to 3 years	90 μg/day
4 to 8 years	150 μg/day
9 to 13 years	280 μg/day
14 to 18 years	400 μg/day

Thus, the National Academy of Sciences ULs for pre-adolescents range from 45 μg/day for infants 0–6 months to 280 μg/day for children 9–13 [142]. Overt signs of selenium toxicity, such as brittle hair, have been reported in children exposed to high selenium intake through food in areas such as Punjab, India [138].

Since the WHO drinking water guidelines are supposed to provide adequate protection for all life stages, it is not clear why these age-weight based differences specified by the National Academy of Sciences were not taken into account by the WHO when establishing the drinking-water guideline based on the National Academy of Sciences 400 μg/day UL for adults.

Of additional concern is that the WHO increased the allocation for exposure to selenium in drinking water from 10 to 20 % without providing any references to support this increase [145], a change which doubled the resulting guideline value. This doubling of the allocation to drinking water without providing support from the literature similarly occurred when the WHO revised the guideline for nickel [94, 95].

Finally, since the 2011 WHO drinking water guideline for selenium is based on a 2000 recommendation from the National Academy of Sciences, it does not take into account subsequent studies which found reason to question whether the 400 μg/day UL for total selenium intake or the former WHO guideline of 10 μg/L for selenium in drinking water were sufficiently protective of public health [138–140, 146, 147]. Selenium was originally included in the list of 29 substances contracted for review by WCA Environment Ltd. However, at the 2013 Chemical Working Group Meeting [3], it was noted that Health Canada has recently reviewed selenium, so selenium was dropped from the list of chemicals contracted with WCA Environment Ltd.

## Conclusions

In 2011, the WHO released the 4th edition of its *Guidelines for Drinking-water Quality*, in which the WHO withdrew the guidelines for manganese and molybdenum, suspended the guideline for long-term exposure to nitrite, did not establish a guideline for aluminum despite stating that a health-based guideline could be derived, and raised the guidelines for boron, nickel, uranium, mercury and selenium. For each of these inorganic compounds, a close examination of the WHO’s background documents suggests that the recent decisions may not have taken into account readily available occurrence data or key toxicological research studies from the last decade, may not have followed WHO stated standard practices for deriving guidelines, or made mathematical, or other errors [1].

The minutes of the December 2013 WHO Chemical Aspects Working Group meeting state that the current policy of the WHO is to not establish a formal health-based drinking-water guideline if the calculated guideline is greater than an expected treatability threshold [3]. “A smaller working group needs to be convened to discuss broader issues, including … what to do with the treatment and analytical method annexes for the fifth edition (e.g. it may be better to move away from chemical-specific information, as it gets out of date quickly, and give information on standard analytical methods or types of treatment that can be used for specific chemical groups or classes)” [3]. Given that background documents, summary statements, addendums, and the *Guidelines for Drinking-water Quality* are regularly updated, it is suggested that the most accurate and current chemical-specific information be used to recommend global public health policies.

The WHO drinking-water guidelines apply to all sources of drinking water from large municipalities to individual households, cover both natural and anthropogenic sources of contamination, and are vital for providing guidance to governments, other stakeholders, and researchers. Given the issues raised here, it is recommended that the WHO reconsider its decisions to withdraw, suspend, not establish, or raise its drinking-water guidelines for manganese, molybdenum, nitrite, aluminum, boron, nickel, uranium, mercury, selenium, and possibly other chemicals. It is also important for the WHO to reconsider its plans to revise (or not revise) these guidelines, to update its database on health effects due to chronic exposures to inorganic toxic substances, and to reconsider its timeline for published revisions [3]. In the meantime, stakeholders must note that the current 2011 WHO guidelines may not provide sufficient protection from the adverse health effects associated with chronic exposure to these inorganic toxic substances in drinking water.

## References

[1] World Health Organization (WHO). Guidelines for drinking-water quality*.* 4th ed. Geneva: WHO; 2011. p. xx, 3, 161–162, 164, 177, 183, 223–224, 311, 323–324, 343, 387, 389–390, 394, 398–399, 413–415, 430, 471.

[2] Greenwood NN, Earnshaw A (1989). Chemistry of the elements.

[3] WHO (World Health Organization). World Health Organization meetings on the guidelines for drinking-water quality, 2–5 December 2013, Geneva, Switzerland. 2014. http://www.who.int/entity/water_sanitation_health/dwq/GDWQ_2013_Repor_microbial_aspects.pdf. Accessed June 17 2014.

[4] WHO (World Health Organization). World Health Organization water quality and health joint expert meeting, 18–22 March 2013, Dübendorf, Switzerland. 2013. http://www.who.int/entity/water_sanitation_health/dwq/GDWQ_2013_Meeting_Report.pdf. Accessed December 3 2014.

[5] WHO (World Health Organization). Meeting on the guidelines for drinking-water quality, 5–7 June 2014, Singapore. 2014. http://www.who.int/entity/water_sanitation_health/dwq/Singapore_meeting_report_2014.pdf. Accessed November 11 2014.

[6] Frisbie SH, Mitchell EJ, Dustin H, Maynard DM, Sarkar B (2012). World Health Organization discontinues its drinking-water guideline for manganese. Environ Health Perspect..

[7] WHO (World Health Organization). Manganese in drinking-water: Background document for development of WHO guidelines for drinking-water quality. 2011. http://www.who.int/entity/water_sanitation_health/dwq/chemicals/manganese.pdf. Accessed June 9 2014.

[8] Frisbie SH, Ortega R, Maynard DM, Sarkar B (2002). The concentrations of arsenic and other toxic elements in Bangladesh’s drinking water. Environ Health Perspect.

[9] Frisbie SH, Mitchell EJ, Mastera LJ, Maynard DM, Yusuf AZ, Siddiq MY (2009). Public health strategies for western Bangladesh that address the arsenic, manganese, uranium and other toxic elements in drinking water. Environ Health Perspect..

[10] Bacquart T, Bradshaw K, Frisbie SH, Mitchell EJ, Springston G, Defelice J (2012). A survey of arsenic, manganese, boron, thorium, and other toxic metals in the groundwater of a West Bengal, India neighbourhood. Metallomics..

[11] Bacquart T, Frisbie SH, Mitchell EJ, Grigg L, Cole C, Small C (2015). Multiple inorganic toxic substances contaminating the groundwater of Myingyan Township, Myanmar: Arsenic, manganese, fluoride, iron, and uranium. Sci Total Environ..

[12] Ljung K, Vahter M (2007). Time to re-evaluate the guideline value for manganese in drinking water?. Environ Health Perspect.

[13] Aschner M, Erikson KM, Hernández HE, Tjalkens RB (2009). Manganese and its role in Parkinson’s disease: From transport to neuropathology. Neuromolecular Med.

[14] Bowman AB, Kwakye GF, Hernándezc EH, Aschner M (2011). Role of manganese in neurodegenerative diseases. J Trace Elem Med Biol..

[15] Guilarte TR (2010). Manganese and Parkinson’s disease: a critical review and new findings. Environ Health Perspect.

[16] Kim Y, Bowler RM, Abdelouahab N, Harris M, Gocheva V, Roels HA (2011). Motor function in adults of an Ohio community with environmental manganese exposure. Neurotoxicol..

[17] Lucchini RG, Martin CJ, Doney BC (2009). From manganism to manganese-induced parkinsonism: a conceptual model based on the evolution of exposure. Neuromolecular Med.

[18] Bowler R, Mergler D, Sassine MP, Larribe F, Hudnell HK (1999). Neuropsychiatric effects of manganese on mood. Neurotoxicology.

[19] Ghazali AR, Kamarulzaman F, Normah CD, Ahmad M, Ghazali SE, Ibrahim N (2013). Levels of metallic elements and their potential relationships to cognitive function among elderly from Federal Land Development Authority (FELDA) settlement in Selangor Malaysia. Biol Trace Elem Res..

[20] Kawamura RH, Ikuta S, Fukuzumi S, Yamada R, Tsubaki S (1941). Intoxication by manganese in well water. Kitasato Arch Exp Med..

[21] Kondakis XG, Makris N, Leotsinidis M, Prinou M, Papapetropoulos T (1989). Possible health effects of high manganese concentration in drinking water. Arch Environ Health.

[22] Shohag H, Ullah A, Qusar S, Rahman M, Hasnat A (2012). Alterations of serum zinc, copper, manganese, iron, calcium, and magnesium concentrations and the complexity of interelement relations in patients with obsessive–compulsive disorder. Biol Trace Elem Res..

[23] Solís-Vivanco R, Rodríguez-Agudelo Y, Riojas-Rodríguez H, Ríos C, Rosas I, Montes S (2009). Cognitive impairment in an adult Mexican population non-occupationally exposed to manganese. Environ Toxicol Pharmacol.

[24] Roos PM, Lierhagen S, Flaten TP, Syversen T, Vesterberg O, Nordberg M (2012). Manganese in cerebrospinal fluid and blood plasma of patients with amyotrophic lateral sclerosis. Exp Biol Med. (London, U. K.).

[25] Bouchard M, Laforest F, Vandelac L, Bellinger D, Mergler D (2007). Hair manganese and hyperactive behaviors: pilot study of school-age children exposed through tap water. Environ Health Perspect.

[26] Bouchard MF, Sauvé S, Barbeau B, Legrand M, Brodeur M-È, Bouffard T (2011). Intellectual impairment in school-age children exposed to manganese from drinking water. Environ Health Perspect.

[27] Menezes-Filho JA, Bouchard M, Sarcinelli PN, Moreira JC (2009). Manganese exposure and the neuropsychological effect on children and adolescents: A review. Rev Panam Salud Publica.

[28] Rodríguez-Barranco M, Lacasaña M, Aguilar-Garduño C, Alguacil J, Gil F, González-Alzaga B (2013). Association of arsenic, cadmium and manganese exposure with neurodevelopment and behavioural disorders in children: a systematic review and meta-analysis. Sci Total Environ..

[29] Zoni S, Lucchini RG (2013). Manganese exposure: cognitive, motor and behavioral effects on children: a review of recent findings. Curr Opin Pediatr.

[30] Polańska K, Jurewicz J, Hanke W. Review of current evidence on the impact of pesticides, polychlorinated biphenyls and selected metals on attention deficit/hyperactivity disorder in children. Int J Occupational Med Environ Health. 2013;26(1):16–38. 10.2478/s13382-013-0073-7 23526196

[31] Grandjean P, Landrigan PJ (2014). Neurobehavioural effects of developmental toxicity. Lancet Neurol..

[32] Lin CC, Chen YC, Su FC, Lin CM, Liao HF, Hwang YH (2013). In utero exposure to environmental lead and manganese and neurodevelopment at 2 years of age. Environ Res..

[33] Oulhote Y, Mergler D, Barbeau B, Bellinger DC, Bouffard T, Brodeur MÈ (2014). Neurobehavioral function in school-age children exposed to manganese in drinking water. Environ Health Perspect.

[34] Roberts AL, Lyall K, Hart JE, Laden F, Just AC, Bobb JF (2013). Perinatal air pollutant exposures and autism spectrum disorder in the children of Nurses’ Health Study II participants. Environ Health Perspect.

[35] Torres-Agustín R, Rodríguez-Agudelo Y, Schilmann A, Solís-Vivanco R, Montes S, Riojas-Rodríguez H (2013). Effect of environmental manganese exposure on verbal learning and memory in Mexican children. Environ Res..

[36] Rink SM, Ardoino G, Queirolo EI, Cicariello D, Mañay N, Kordas K (2014). Associations between hair manganese levels and cognitive, language, and motor development in preschool children from Montevideo, Uruguay. Arch Environ Occupational Health..

[37] Abdullah MM, Ly AR, Goldberg WA, Clarke-Stewart KA, Dudgeon JV (2012). Heavy metal in children’s tooth enamel: related to autism and disruptive behaviors?. J Autism Dev Disord..

[38] Vigeh M, Yokoyama K, Ramezanzadeh F, Dahaghin M, Sakai T, Morita Y (2006). Lead and other trace metals in preeclampsia: a case–control study in Tehran. Iran. Environ Res..

[39] Basu R, Harris M, Sie L, Malig B, Broadwin R, Green R (2014). Effects of fine particulate matter and its constituents on low birth weight among full-term infants in California. Environ Res..

[40] Gražulevičiene R, Nadisauskiene R, Buinauskiene J, Grazulevicius T (2009). Effects of elevated levels of manganese and iron in drinking water on birth outcomes. Polish J Environ Stud.

[41] Eum JH, Cheong HK, Ha EH, Ha M, Kim Y, Hong YC (2014). Maternal blood manganese level and birth weight: A MOCEH birth cohort study. Environ Res..

[42] Guan H, Wang M, Li X, Piao F, Li Q, Xu L (2014). Manganese concentrations in maternal and umbilical cord bold: related to birth size and environmental factors. Eur J Public Health.

[43] Zota AR, Ettinger AS, Bouchard M, Amarasiriwardena C, Schwartz J, Hu H (2009). Maternal blood manganese levels and infant birth weight. Epidemiology.

[44] Yu XD, Cao LL, Yu XG (2013). Elevated cord serum manganese level is associated with a neonatal high ponderal index. Environ Res..

[45] Yazbeck C, Moreau T, Sahuquillo J, Takser L, Huel G (2006). Effect of maternal manganese blood levels on erythrocyte calcium-pump activity in newborns. Sci Total Environ..

[46] Hafeman D, Factor-Litvak P, Cheng Z, van Geen A, Ahsan H (2007). Association between manganese exposure through drinking water and infant mortality in Bangladesh. Environ Health Perspect.

[47] Spangler AH, Spangler JG (2009). Groundwater manganese and infant mortality rate by county in North Carolina: an ecological analysis. Ecohealth.

[48] Aschengrau A, Zierler S, Cohen A (1989). Quality of community drinking water and the occurrence of spontaneous abortion. Arch Environ Health.

[49] Cherry N, Shaik K, McDonald C, Chowdhury Z (2010). Manganese, arsenic, and infant mortality in Bangladesh: an ecological analysis. Arch Environ Occupational Health.

[50] Rahman SM, Åkesson A, Kippler M, Grandér M, Hamadani JD, Streatfield PK (2013). Elevated manganese concentrations in drinking water may be beneficial for fetal survival. PLOS ONE.

[51] Li P, Zhong Y, Jiang X, Wang C, Zuo Z, Sha A (2012). Seminal plasma metals concentration with respect to semen quality. Biol Trace Elem Res..

[52] Li Y, Wu J, Zhou W, Gao E (2012). Effects of manganese on routine semen quality parameters: results from a population-based study in China. BMC Public Health..

[53] Zeng Q, Zhou B, Feng W, Wang YX, Liu AL, Yue J (2013). Associations of urinary metal concentrations and circulating testosterone in Chinese men. Reprod Toxicol..

[54] Takser L, Mergler D, de Grosbois S, Smargiassi A, Lafond J (2004). Blood manganese content at birth and cord serum prolactin levels. Neurotoxicol Teratol..

[55] Montes S, Schilmann A, Riojas-Rodriguez H, Rodriguez-Agudelo Y, Solis-Vivanco R, Rodriguez-Dozal SL (2011). Serum prolactin rises in Mexican school children exposed to airborne manganese. Environ Res..

[56] Giray B, Arnaud J, Sayek İ, Favier A, Hıncal F (2010). Trace elements status in multinodular goiter. J Trace Elem Med Biol..

[57] Savchenko OV, Toupeleev PA (2012). Lead, cadmium, manganese, cobalt, zinc and copper levels in whole blood of urban teenagers with non-toxic diffuse goiter. Int J Environ Health Res.

[58] Oulhote Y, Mergler D, Bouchard MF (2014). Sex- and age-differences in blood manganese levels in the U.S. general population: national health and nutrition examination survey 2011–2012. Environ Health..

[59] Brna P, Gordon K, Dooley JM, Price V (2011). Manganese toxicity in a child with iron deficiency and polycythemia. J Child Neurol.

[60] Rahman MA, Rahman B, Ahmed N (2013). High blood manganese in iron-deficient children in Karachi. Public Health Nutr.

[61] Wood RJ (2009). Manganese and birth outcome. Nutr Rev.

[62] Sahni V, Léger Y, Panaro L, Allen M, Giffin S, Fury D (2007). Case report: A metabolic disorder presenting as pediatric manganism. Environ Health Perspect.

[63] Hernandez EH, Discalzi G, Dassi P, Jarre L, Pira E (2003). Manganese intoxication: the cause of an inexplicable epileptic syndrome in a 3 year old child. Neurotoxicol..

[64] Taba P (2013). Metals and movement disorders. Curr Opin Neurol.

[65] World Health Organization (WHO) (2004). Guidelines for drinking-water quality.

[66] World Health Organization (WHO) (1996). Guidelines for drinking-water quality. Volume 2: Health criteria and other supporting information.

[67] WHO (World Health Organization). Molybdenum in drinking-water: Background document for development of WHO guidelines for drinking-water quality. 2011. http://www.who.int/entity/water_sanitation_health/dwq/chemicals/en/molybdenum.pdf. Accessed May 30 2014.

[68] Groschen GE, Arnold TL, Morrow WS, Warner KL. United States Geological Survey Scientific Investigations Report 2009–5006: Occurrence and distribution of iron, manganese, and selected trace elements in ground water in the glacial aquifer system of the northern United States. 2008. http://pubs.usgs.gov/sir/2009/5006/pdf/SIR2009-5006.pdf. Accessed October 28 2011.

[69] Senior LA, Sloto RA. Arsenic, boron, and fluoride concentrations in ground water in and near diabase intrusions, Newark Basin, Southeastern Pennsylvania. USGS Scientific Investigations Report 2006–5261. 2006. http://pubs.usgs.gov/sir/2006/5261/pdf/sir2006-5261.pdf. Accessed December 11 2008.

[70] Bertini LM, Cohen IM, Resnizky SM, Gomez CD (1993). Contribution of neuron activation analysis to a geochemical study of contamination by arsenic, antimony, selenium, and molybdenum. J Radioanalytical Nucl Chem.

[71] Galindo G, Sainato C, Dapeña C, Fernández-Turiel JL, Gimeno D, Pomposiello MC (2007). Surface and groundwater quality in the northeastern region of Buenos Aires Province, Argentina. J S Am Earth Sci..

[72] Smedley PL, Nicolli HB, Macdonald DMJ, Barros AJ, Tullio JO (2002). Hydrogeochemistry of arsenic and other inorganic constituents in groundwaters from La Pampa, Argentina. Appl Geochem..

[73] Smedley PL, Knudsen J, Maiga D (2007). Arsenic in groundwater from mineralised Proterozoic basement rocks of Burkina Faso. Appl Geochem..

[74] Feldman PR, Rosenboom JW, Saray M, Navuth P, Samnang C, Iddings S (2007). Assessment of the chemical quality of drinking water in Cambodia. J Water Health.

[75] Rango T, Kravchenko J, Atlaw B, McCornick PG, Jeuland M, Merola B (2012). Groundwater quality and its health impact: An assessment of dental fluorosis in rural inhabitants of the Main Ethiopian Rift. Environ Int..

[76] Reimann C, Bjorvatn K, Tekle-Haimanot R, Melaku Z, Siewers U (2000). Drinking water quality.

[77] Bidhendi GRN, Karbassi AR, Nasrabadi T, Hoveidi H (2007). Influence of copper mine on surface water quality. Int J Environ Sci Tech.

[78] WHO (World Health Organization). Nitrate and nitrite in drinking-water: Background document for development of WHO guidelines for drinking-water quality. 2011. http://www.who.int/entity/water_sanitation_health/dwq/chemicals/nitratenitrite_background.pdf. Accessed May 30 2014.

[79] Islam MR, Salminen R, Lahermo PW (2000). Arsenic and other toxic elemental contaminzation of groundwater, surface water, and soil in Bangladesh and its possible effects on human health. Environ Geochem Health..

[80] Miranda RG, Pereira SFP, Alves DTV, Oliveira GRF. Quality of water resources in the Amazon region - Rio Tapajós: Assessing the case for chemical elements and physical-chemical parameters. Revista Ambiente & Água 2009, 4(2). http://dx.doi.org/10.4136/ambi-agua.88.

[81] Suassuna K. Contamination in Paulínia by aldrin, dieldrin, endrin and other toxic chemicals produced and disposed of by Shell Chemicals of Brazil. Toxic Substances and Technologies Campaign Greenpeace Brazil. 2001. http://www.greenpeace.org/international/Global/international/planet-2/report/2001/4/contamination-in-paul-nia-by-a.pdf. Accessed October 26 2011.

[82] Kortatsi BK. Groundwater quality in the Wassa West District of the Western Region of Ghana. W Afr J Appl Ecol 2007, 11(1). http://www.ajol.info/index.php/wajae/article/download/45729/29207.

[83] Mukherjee-Goswami A, Nath B, Jana J, Sahu SJ, Sarkar MJ, Jacks G (2008). Hydrogeochemical behavior of arsenic-enriched groundwater in the deltaic environment: Comparison between two study sites in West Bengal, India. J Contam Hydrol..

[84] Berisha F, Goessler W. Investigation of drinking water quality in Kosovo. J Environ Public Health 2013. http://dx.doi.org/10.1155/2013/374954. 10.1155/2013/374954 PMC3595666 23509472

[85] Pullanikkatil D. Water quality assessment of Mohokare River. Lesotho: Department of Civil Engineering, Lerotholi Polytechnic; 2008. http://www.waternetonline.ihe.nl/downloads/uploads/symposium/zambia-2007/Water and Environment/Pullanikkatil.pdf. Accessed October 30 2011.

[86] IGES (Institute for Global Environmental Strategies), IGES (2013). The study of the management of groundwater resources in Sri Lanka. Sustainable groundwater management in Asian cities: A summary report of research on sustainable water management in Asia.

[87] Rosborg I, Nihlgård B, Gerhardsson L (2003). Inorganic constituents of well water in one acid and one alkaline area of south Sweden. Water Air Soil Pollut..

[88] Bowell RJ, McEldowney S, Warren A, Mathew B, Bwankuzo M (1996). Biogeochemical factors affecting groundwater quality in central Tanzania. Environ Geochem Health..

[89] Bakar C, Karaman HI, Baba A, Şengünalp F (2010). Effect of high aluminum concentration in water resources on human health, case study: Biga Peninsula, northwest part of Turkey. Arch Environ Contam Toxicol..

[90] BGS (British Geological Survey). Groundwater quality: Uganda. 2001. http://www.wateraid.org/~/media/Publications/groundwater-quality-information-uganda.pdf. Accessed October 30 2011.

[91] WHO (World Health Organization). Aluminium in drinking-water: Background document for development of WHO guidelines for drinking-water quality. 2010. http://www.who.int/entity/water_sanitation_health/publications/aluminium.pdf. Accessed May 29 2014.

[92] WHO (World Health Organization). Boron in drinking-water: Background document for development of WHO guidelines for drinking-water quality. 2009. http://whqlibdoc.who.int/hq/2009/WHO_HSE_WSH_09.01_2_eng.pdf. Accessed October 13 2011.

[93] World Health Organization (WHO) (2006). Guidelines for drinking-water quality. First addendum to the third edition.

[94] World Health Organization (WHO) (1998). Guidelines for drinking-water quality. Addendum to volume 2: Health criteria and other supporting information.

[95] WHO (World Health Organization). Nickel in drinking-water: Background document for development of WHO guidelines for drinking-water quality. 2005. http://www.who.int/water_sanitation_health/dwq/chemicals/nickeladd270605.pdf. Accessed April 30 2013.

[96] Frisbie SH, Mitchell EJ, Sarkar B (2013). World Health Organization increases its drinking-water guideline for uranium. Environ Sci Processes Impacts..

[97] WHO (World Health Organization). Uranium in drinking-water: Background document for development of WHO guidelines for drinking-water quality. 2011. http://www.who.int/water_sanitation_health/dwq/chemicals/uranium_forcomment_20110211_en.pdf. Accessed October 13 2011.

[98] Kurttio P, Harmoinen A, Saha H, Salonen L, Karpas Z, Komulainen H (2006). Kidney toxicity of ingested uranium from drinking water. Am J Kidney Dis.

[99] Kurttio P, Auvinen A, Salonen L, Saha H, Pekkanen J, Mäkeläinen I (2002). Renal effects of uranium in drinking water. Environ Health Perspect.

[100] Kurttio P, Komulainen H, Leino A, Salonen L, Auvinen A, Saha H (2005). Bone as a possible target of chemical toxicity of natural uranium in drinking water. Environ Health Perspect.

[101] Ott MG, Langner RR, Holder BB (1975). Vinyl chloride exposure in a controlled industrial environment. Arch Environ Health.

[102] Tsai SY, Chou HY, The HW, Chen CM, Chen CJ (2003). The effects of chronic arsenic exposure from drinking water on the neurobehavioral development in adolescence. Neurotoxicology.

[103] WHO (World Health Organization). Selenium in drinking-water: Background document for development of WHO guidelines for drinking-water quality. 2003. http://apps.who.int/iris/bitstream/10665/75424/1/WHO_SDE_WSH_03.04_13_eng.pdf. Accessed October 21 2014.

[104] WHO (World Health Organization). Mercury in drinking-water: Background document for development of WHO guidelines for drinking-water quality. 2005. http://www.who.int/water_sanitation_health/dwq/chemicals/mercuryfinal.pdf. Accessed May 29 2013.

[105] WHO (World Health Organization). Mercury in drinking-water: Background document for development of WHO guidelines for drinking-water quality. 2003. http://www.who.int/water_sanitation_health/dwq/chemicals/en/mercury.pdf. Accessed December 30 2014.

[106] JECFA (Joint Food and Agriculture Organization/World Health Organization Expert Committee on Food Additives). Evaluation of mercury, lead, cadmium and the food additives amaranth, diethylpyrocarbonate, and octyl gallate. 1972. http://www.inchem.org/documents/jecfa/jecmono/v004je02.htm. Accessed July 18 2014.

[107] JECFA (Joint Food and Agriculture Organization/World Health Organization Expert Committee on Food Additives). Evaluation of certain food additives and contaminants. 2004. http://whqlibdoc.who.int/trs/WHO_TRS_922.pdf. Accessed July 18 2014.

[108] JECFA (Joint Food and Agriculture Organization/World Health Organization Expert Committee on Food Additives). Evaluation of certain food additives and contaminants. 2007. http://whqlibdoc.who.int/trs/WHO_TRS_940_eng.pdf. Accessed July 23 2014.

[109] IPCS (International Programme on Chemical Safety). Elemental mercury and inorganic mercury compounds: Human health aspects. 2003. http://www.who.int/ipcs/publications/cicad/en/cicad50.pdf. Accessed April 18 2013.

[110] JECFA (Joint Food and Agriculture Organization/World Health Organization Expert Committee on Food Additives). Evaluation of certain food additives and contaminants. 2011. http://whqlibdoc.who.int/trs/who_trs_959_eng.pdf. Accessed July 23 2014.

[111] Morita M, Yoshinaga J, Edmondst JS (1998). The determination of mercury species in environmental and biological samples. Pure Appl Chem..

[112] Yu LP, Yan XP (2003). Factors affecting the stability of inorganic and methylmercury during sample storage. Trends Anal Chem.

[113] Wickre JB, Folt CL, Sturup S, Karagas MR (2004). Environmental exposure and fingernail analysis of arsenic and mercury in children and adults in a Nicaraguan gold mining community. Arch Environ Health.

[114] Ishaq M, Jan FA, Khan MA, Ihsanullah I, Ahmad I, Shakirullah M (2013). Effect of mercury and arsenic from industrial effluents on the drinking water and comparison of the water quality of polluted and non-polluted areas: A case study of Peshawar and Lower Dir. Environ Monit Assess..

[115] Maramba NPC, Reyes JP, Francisco-Rivera AT, Panganiban LCR, Dioquino C, Dando N (2006). Environmental and human exposure assessment monitoring of communities near an abandoned mercury mine in the Philippines: A toxic legacy. J Environ Manage..

[116] Kowalski A, Siepak M, Boszke L (2007). Mercury contamination of surface and ground waters of Poznań, Poland. Polish J of Environ Stud..

[117] Murphy EA, Dooley J, Windom HL, Smith RG (1994). Mercury species in potable ground water in southern New Jersey. Water Air Soil Pollut..

[118] Zhang H, Feng X, Larssen T, Shang L, Vogt RD, Lin Y (2010). Fractionation, distribution and transport of mercury in rivers and tributaries around Wanshan Hg mining district, Guizhou Province, Southwestern China: Part 2 – methylmercury. Appl Geochem.

[119] Donkor AK, Bonzongo JC, Nartey VK, Adotey DK (2006). Mercury in different environmental compartments of the Pra River Basin, Ghana. Sci Total Environ..

[120] Karunasagar D, Balarama Krishna MV, Anjaneyulu Y, Arunachalam J (2006). Studies of mercury pollution in a lake due to a thermometer factory situated in a tourist resort: Kodaikkanal, India. Environ Pollut..

[121] Bravo AG, Cosio C, Amouroux D, Zopfi J, Chevalley PA, Spangenberg JE (2014). Extremely elevated methyl mercury levels in water, sediment and organisms in a Romanian reservoir affected by release of mercury from a chlor-alkali plant. Water Res..

[122] Regnell O, Hammar T, Helgee A, Troedsson B (2001). Effects of anoxia and sulfide on concentrations of total and methyl mercury in sediment and water in two Hg-polluted lakes. Can J Fish Aquat Sci.

[123] Holmes P, James KAF, Levy LS (2009). Is low-level environmental mercury exposure of concern to human health?. Sci Total Environ..

[124] Carocci A, Rovito N, Sinicropi MS, Genchi G, Whitacre DM (2014). Mercury toxicity and neurodegenerative effects. Reviews of environmental contamination and toxicology.

[125] NTP (National Toxicology Program). Toxicology and carcinogenesis studies of mercuric chloride in F344 rats and B6C3F1 mice (CAS No. 7487-94-7). 1993. http://ntp.niehs.nih.gov/ntp/htdocs/lt_rpts/tr408.pdf. Accessed June 30 2014. 12621522

[126] Azevedo BF, Furieri LB, Pecanha FM, Wiggers GA, Vassallo PF, Simoes MR et al.. Toxic effects of mercury on the cardiovascular and central nervous systems. J Biomed Biotechnol 2012. http://dx.doi.org/10.1155/2012/949048. 10.1155/2012/949048 PMC3395437 22811600

[127] El-Sherbeeny AM, Odom JV, Smith JE (2006). Visual system manifestations due to systemic exposure to mercury. Cutan Ocul Toxicol..

[128] Hoshino ACH, Ferreira HP, Malm O, Carvallo RM, Câmara VM (2012). A systematic review of mercury ototoxicity. Cad Saúde Pública.

[129] Tan SW, Meiller JC, Mahaffey KR (2009). The endocrine effects of mercury in humans and wildlife. Crit Rev Toxicol.

[130] Wirth JJ, Mijal RS (2010). Adverse effects of low level heavy metal exposure on male reproductive function. Syst Biol Reprod Med..

[131] Bose-O’Reilly S, McCarty KM, Steckling N, Lettmeier B (2010). Mercury exposure and children’s health. Curr Probl Pediatr Adolesc Health Care.

[132] Hightower JM, Moore D (2003). Mercury levels in high-end consumers of fish. Environ Health Perspect.

[133] IPCS (International Programme on Chemical Safety). Environmental health criteria 101: Methylmercury. 1990. http://www.inchem.org/documents/ehc/ehc/ehc101.htm. Accessed July 2 2014.

[134] Ronchetti R, Zuurbier M, Jesenak M, Koppe JG, Ahmed UF, Ceccatelli S (2006). Children’s health and mercury exposure. Acta Paediatr.

[135] U.S. EPA (United States Environmental Protection Agency). Water quality criterion for the protection of human health: Methylmercury. 2001. http://www.waterboards.ca.gov/water_issues/programs/tmdl/records/state_board/2008/ref2664.pdf. Accessed December 1 2014.

[136] U.S. EPA (United States Environmental Protection Agency). Technical support document for action on the state of Oregon’s new and revised human health water quality criteria for toxics and associated implementation provisions submitted July 12 and 21, 2011. 2011. http://www.epa.gov/region10/pdf/water/or-tsd-hhwqs-2011.pdf. Accessed December 1 2014.

[137] Watras CJ, Morrison KA, Host JS (1995). Concentration of mercury species in relationship to other site-specific factors in the surface waters of northern Wisconsin lakes. Limnol Oceanogr.

[138] Fairweather-Tait SJ, Bao Y, Broadley MR, Collings R, Ford D, Hesketh JE (2011). Selenium in human health and disease. Antioxid Redox Signal.

[139] Vinceti M, Bonvicini F, Rothman KJ, Vescovi L, Wang F (2010). The relation between amyotrophic lateral sclerosis and inorganic selenium in drinking water: A population-based case–control study. Environ Health..

[140] Vinceti M, Crespi CM, Malagoli C, Bottecchi I, Ferrari A, Sieri S (2012). A case–control study of the risk of cutaneous melanoma associated with three selenium exposure indicators. Tumori.

[141] Longnecker MP, Taylor PR, Levander OA, Howe SM, Veillon C, McAdam PA (1991). Selenium in diet, blood, and toenails in relation to human health in a seleniferous area. Am J Clin Nutr..

[142] National Academy of Sciences (NAS) (2000). Dietary reference intakes for vitamin C, vitamin E, selenium, and carotenoids.

[143] World Health Organization (WHO) (2004). Food and Agriculture Organization of the United Nations (FAO). Vitamin and mineral requirements in human nutrition.

[144] Yang G, Zhou R (1994). Further observations on the human maximum safe dietary selenium intake in a seleniferous area of China. J Trace Elem Electrolytes Health Dis..

[145] WHO (World Health Organization). Selenium in drinking-water: Background document for development of WHO guidelines for drinking-water quality. 2011. http://www.who.int/entity/water_sanitation_health/dwq/chemicals/selenium.pdf. Accessed September 6 2014.

[146] Stranges S, Marshall JR, Natarajan R, Donahue RP, Trevisan M, Combs GF (2007). Effects of long-term selenium supplementation on the incidence of type 2 diabetes. Ann Intern Med..

[147] Vinceti M, Maraldi T, Bergomi M, Malagoli C (2009). Risk of chronic low-dose selenium overexposure in humans: Insights from epidemiology and biochemistry. Rev Environ Health.

